# Glypican-3-Expressing Hepatocellular Carcinoma in a Non-Cirrhotic Patient with Nonalcoholic Steatohepatitis: Case Report and Literature Review

**DOI:** 10.4021/gr224w

**Published:** 2010-09-20

**Authors:** Mona H. Ismail

**Affiliations:** a College of Medicine, University of Dammam, Consultant Hepatologist and Gastroenterologist, Division of Gastroenterology, Department of Internal Medicine, King Fahad Hospital of the University, Saudi Arabia; b Department of Internal Medicine, Division of Gastroenterology & Multiorgan Transplant Programme, King Fahad Specialist Hospital, Dammam, Saudi Arabia

**Keywords:** Hepatocellular carcinoma, Nonalcoholic steatohepatitis, Glypican-3, Noncirrhotic

## Abstract

Hepatocellular carcinoma (HCC) is one of the most common cancers worldwide. Nonalcoholic steatohepatitis (NASH) is now considered as the major cause of cryptogenic cirrhosis, which can progress to HCC. Glypican-3 is a member of the Heparan Sulfate Proteoglycan (HSP) family that plays a role in cell growth, differentiation, and migration. Glypican-3 is significantly up-regulated in a majority of HCCs compared to normal and benign liver samples. Glypican-3 protein is detectable in around 40-53% of HCC patients whereas it is not detectable in the serum of healthy individuals. There are several reports of HCC arising in the setting of non-cirrhotic NASH. This report describes a case of HCC that expressed Glypican-3 and arose in a 47-year-old female with noncirrhotic NASH.

## Introduction

Hepatocellular carcinoma (HCC) is a common and deadly malignancy and its incidence is increasing [1]. Most cases of HCC develop in the presence of advanced chronic liver disease, related mainly to the hepatitis B and C viruses and alcohol abuse, in which ongoing liver injury and regeneration predispose patients to neoplasia over time [2]. The major etiologies and risk factors for HCC development are well-defined, and some of the multiple steps involved in hepatocarcinogenesis have been elucidated in recent years [3]. Nonalcoholic steatohepatitis (NASH) is now considered as the major cause of cryptogenic cirrhosis in many regions of the world. Progression of NASH to cirrhosis and hepatocellular damage is typically slow, and most cases of HCC arise in patients with preexisting cirrhosis. The majority of patients with “cryptogenic” cirrhosis complicated by HCC have risk factors for NASH [4]. Unfortunately, the lack of steatosis and necroinflammation once cirrhosis occurs provides little evidence for the etiology of HCC [5]. Advances in the diagnosis and management of HCC have significant impact in patients who are at risk of developing HCC. Serological markers for HCC are important for early diagnosis and for monitoring tumor aggressiveness, treatment responsiveness, recurrence and survival [6]. Surveillance of patients at risk of developing HCC is based on ultrasound (US) examinations and serum alpha fetoprotein (AFP) performed at either 6 or 12 months interval. Early detection of HCC in patients with cirrhosis is a challenging issue with certain limitations. For example, certain subtypes of HCC are not associated with an elevated serum AFP level [7]. Imaging studies such as US and computerized tomography (CT) standard imaging techniques play some role in the identification and localisation of HCC. However, the diagnostic sensitivity of these imaging modalities decreases in small lesions, especially in those with size less than 2 cm. Thus, the gold standard for diagnosing HCC is pathological examination of the liver tissue [8].

Lately, a novel marker, glypican-3 (GPC3), has been proposed as a diagnostic marker for HCC [9]. GPC3 expression was shown to be markedly elevated in a large proportion of HCCs, whereas preneo­plastic and nonneoplastic liver tissue samples revealed less frequent and no GPC3 expression [10, 11].

There are several reports of HCC arising in the setting of non-cirrhotic NASH [12-14]. This report describes a case of HCC that expressed Glypican-3 and arose in a 47-year-old female with noncirrhotic NASH.

## Case Report

A 49-year-old Saudi woman, who was experiencing right-upper-quadrant pain, was referred by a private clinic for further management after an US revealed a large liver mass. She did not report a history of exposure known to be associated with the development of HCC. Her medical history included type 2 diabetes. She had no risk factors for viral hepatitis and never consumed alcohol. Her sister died at age 50 of an unknown liver disease. Her medications included only Metformin and insulin.

At admission, she was obese (BMI 31 kg/m^2^) with mild hepatomegaly and no evidence of chronic liver disease. There were no ascites or lower limb edema. Laboratory studies revealed the following values: total bilirubin, 0.2 (0.1 - 1.0 mg/dL); alanine transferase, 73 (20 - 65U/L); aspartate aminotransferase, 65 (7 - 41U/L); GGTP, 709 (5 - 85 U/L); total protein, 6.5 (6 - 8g /dL); albumin, 3.2 (3.5 - 4.8 g/dL); platelets, 202,000 (140 - 440 per uL); prothrombin time/international normalized ratio, 0.9; α-fetoprotein level, 10.7 (0 - 15 ng/mL); fasting blood sugar, 297 (70 - 110 mg/dl) and hemoglobin A1c, 8 (4 - 6). Autoimmune (antinuclear antibody and smooth muscle antibody) and hepatitis B and C serology were negative. The gastroscopy was negative for varices.

**Figure 1:**
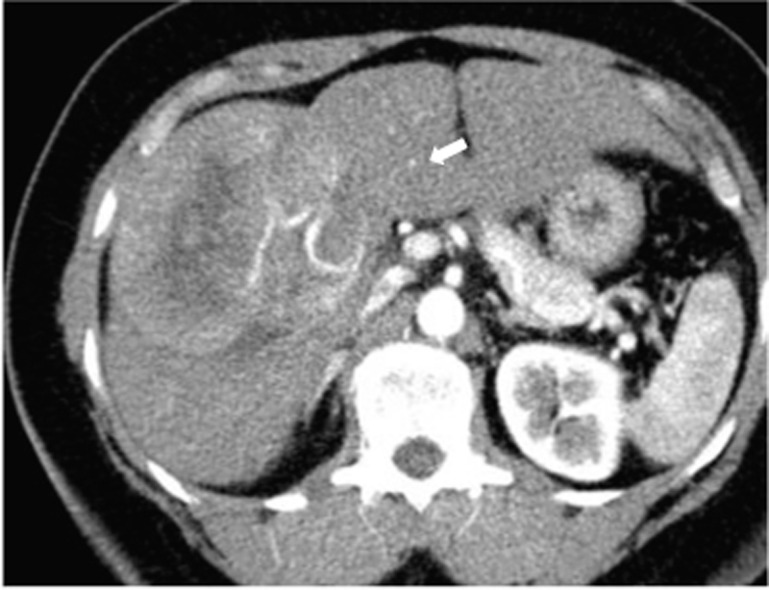
Triphasic CT (Arterial Phase): Enhanced tumor (arrow) with hypodense center and fatty liver.

A triple-phase CT scan showed a 9 x 9 cm mass in segments V/VI of the liver and two smaller lesions in segments III/IV with arterial enhancement, portal venous washout consistent with multifocal HCC, as well as hepatic steatosis (Fig.1). There were no signs of portal hypertension or cirrhosis. The liver biopsy confirmed the diagnosis of HCC, and the patient then underwent resection of liver segments III and V/VI and intraoperative radiofrequency of the remaining lesion.

**Figure 2:**
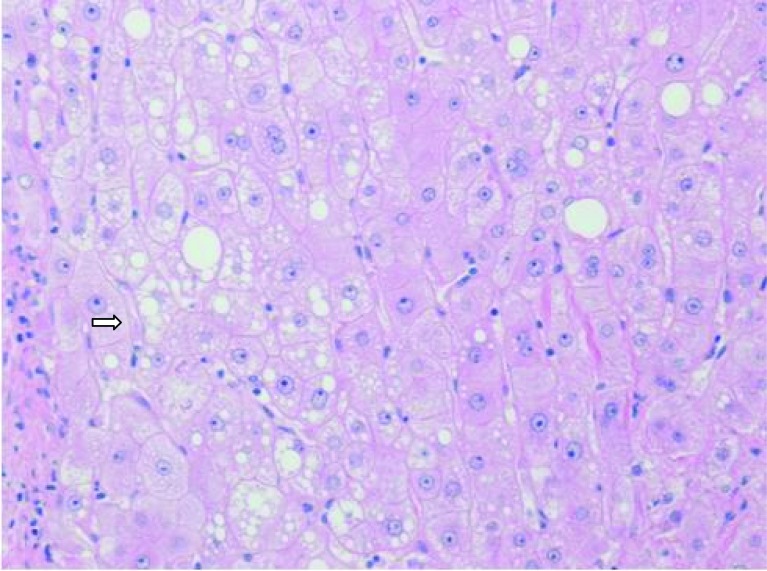
Well-differentiated HCC with micro-steatosis (arrow) (H & E x100).

**Figure 3:**
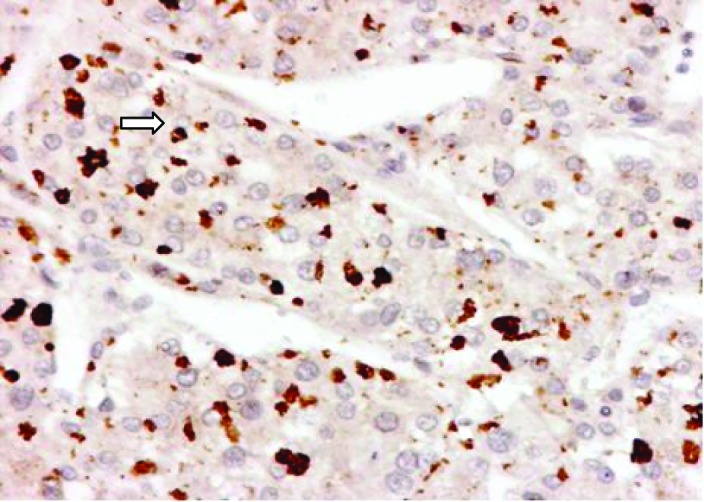
P62 expression of intracytoplasmic hyaline bodies (IHB) (arrow) in Hepatocellular Carcinoma.

**Figure 4:**
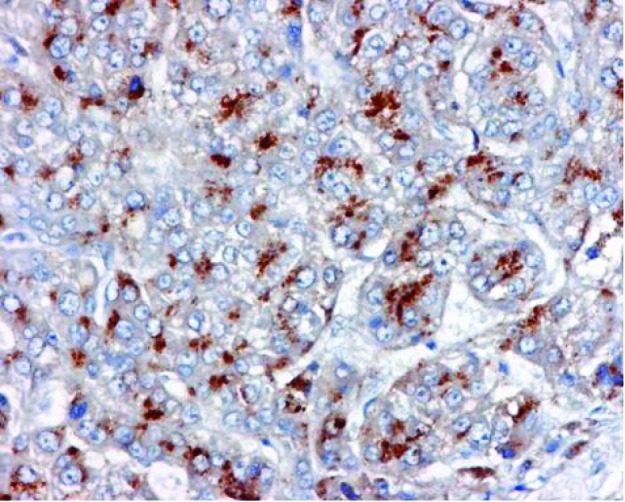
Glypican-3 (GPC-3) expression in Hepatocellular Carcinoma.

**Figure 5:**
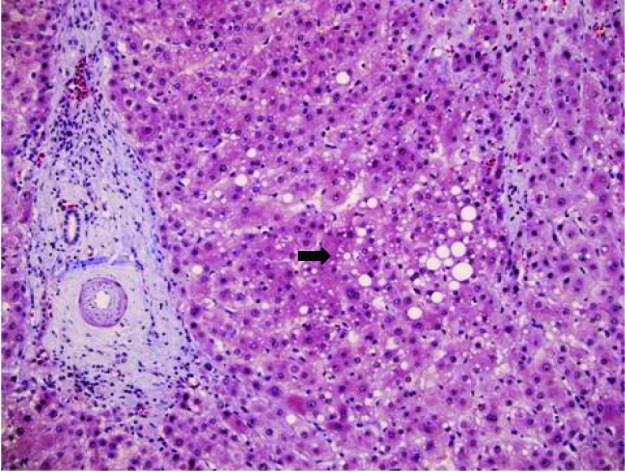
Trichrome stain of liver showing micro-steatosis (arrow) and minimal fibrosis.

The histology revealed well-differentiated, trabecular HCCs with acinar and pseudoacinar patterns and Mallory hyaline bodies. Macrovesicular steatosis was noted in the patient’s tumor cells (Fig. 2), which stained positively for intracytoplasmic hyaline bodies (IHB) (Fig. 3) and had positive Glypican-3 staining (Fig. 4). The sections from the uninvolved liver showed moderate macro- and micro-vesicular steatosis, mild lobular inflammation with infiltration of mixed inflammatory cells and Mallory hyaline. Trichrome staining demonstrated minimal fibrosis (Fig. 5). There was no histopathological evidence for viral hepatitis, α1-antitrypsin deficiency, or hemochromatosis. The hepatitis B core antigen immunostaining, periodic acid Schiff/diastase (PAS/D), and iron histochemistry findings were negative.

Postoperatively, the patient progressively developed signs of liver failure (hyperbilirubinemia and ascites). Follow-up liver US showed the local recurrence of multiple tumors, and the patient was not a candidate for transarterial chemoembolization or Sorafenib. Despite medical care, the patient died six months later from sepsis.

## Discussion

HCC is usually asymptomatic in the early stages. Because of their rapid growth rate and the lack of accurate ways of diagnosis in the early stages, the prognosis and the survival rate for liver cancer patients remains poor. Currently, US, CT, magnetic resonance imaging (MRI) of the liver and in some cases histopathological examination for tumor biopsy is used for diagnosis of HCC with variable costs [15]. The imaging-based diagnosis of small tumors is relatively inaccurate, as cirrhotic and dysplastic nodules mimic HCC radiologically.

The availability of a suitable serological marker to distinguish between HCC and benign liver lesions would, therefore, be very useful for early diagnosis [16]. The only serological marker currently widely used for the diagnosis of HCC is AFP but has limited sensitivity. For example, serum AFP at a cut-off value of 20 ng/mL shows 60-80% sensitivity, although this sensitivity decreases to approximately 20-40% for the detection of small tumors [17]. Also certain subtypes of HCC are not associated with an elevated serum AFP level [6].

GPC3, oncofetal gene over-expressed specifically in human HCC, is a member of the HSP family associated with the cell membrane and the extracellular matrix that plays a role in cell growth, differentiation, and migration [18]. GPC3 promotes the growth of HCC by stimulating the autocrine/paracrine canonical Wnt signaling [19]. Several laboratories have recently reported that Glypican-3 is expressed by a large proportion of HCCs, but is undetectable in normal hepatocytes and non-malignant liver disease [20, 21]. Capurro et al [9] demonstrated that GPC3 immunoassay had potential as a serum diagnostic marker of HCC, with serological sensitivity and specificity of 53% and 95%, respectively. Hippo and colleagues [22] found that serum levels of soluble GPC3, the NH2-terminal portion of GPC3, were significantly higher in HCC patients, compared to patients with cirrhosis and healthy controls. Although the specificity of the test was very high in patients with chronic liver disease, the sensitivity was limited (within the same range as AFP) [23]. The combined use of serum GPC3 and AFP provides a potentially promising tool for diagnosis of HCC [24].

NASH-related cirrhosis is now a well-recognized cause of HCC. The natural history of HCC is fairly long, and the mechanisms of pathogenesis of HCC in the non-fibrotic liver are still unclear. NASH-associated insulin resistance causes inhibition of hepatic mitochondrial fatty acid oxidation; increased intracellular fatty acids may then lead to oxidative DNA damage by stimulating microsomal peroxidases. The exact mechanism behind the development of HCC in NASH remains unclear, although the pathophysiologic mechanisms behind the development of NASH related to insulin resistance and the subsequent inflammatory cascade likely contribute to the carcinogenic potential of NASH [25].

Multiple case reports of HCC in the setting of NASH have been published and this is nicely reviewed by Page et al [26]. The patients were typically male with advanced age at presentation than patients with HCC related to other chronic liver diseases. The risks for HCC development were cirrhosis, diabetes, and obesity. Few patients presented with HCC in the absence of cirrhosis raising the possibility that carcinogenesis can occur in NAFLD in the absence of advanced liver disease [14, 27]. Beale et al [28] conducted a cross sectional study assessing comparing the efficacy of Prothrombin Induced by Vitamin K Absence (PIVKA-II), GCP-3, Squamous Cell Carcinoma Antigen -1 (SCCA-1) and Follistatin, a monomeric protein overexpressed in rat and human liver tumours and reportedly contributing to hepatocarcinogenesis by the inhibition of activins, for the diagnosis of HCC arising on a background of 50 cirrhotic patients (31 had alcoholic liver disease (ALD) and 19 had NAFLD). The serum samples were compared to an independent group of 41 patients with biopsy proven ALD or NAFLD cirrhosis. Serum levels of GP3, SCCA-1 and follistatin had no HCC surveillance benefit in these patients. AFP and PIVKAII were superior to the other markers, particularly in combination. They conclude that while novel means of surveillance are urgently required, the combination of AFP and PIVKAII for HCC is an improvement on AFP alone in ALD/NAFLD patients.

Our patient had no evidence of cirrhosis and to our knowledge, this is the first report of GPC3 expression of HCC in a noncirrhotic NASH, lending support to the potential role and usefulness of GPC3 in diagnosing HCC in this subgroup of patients.

In conclusion, NASH with HCC is not limited to patients with NASH-related cirrhosis, and we describe a case where GPC3 is expressed in a HCC tumor in a patient without frank cirrhosis. The increasing prevalence of NASH, in addition to diabetes and obesity, highlight the need for further cost effectiveness studies on screening for HCC in this population.
